# A Patient With Failed Liver Transplantation After the Use of PD-1 Blockade Combined With Lenvaxen

**DOI:** 10.3389/fmed.2022.712466

**Published:** 2022-02-21

**Authors:** Jun Yin, Meng Wen, Jun Cheng, Lifen Hu, Li Yang, Xiao Chang, Zhongsong Zhou, Hongbin Li, Yan Liu, Jiabin Li

**Affiliations:** ^1^Department of Infectious Diseases, The First Affiliated Hospital of Anhui Medical University, Hefei, China; ^2^Anhui Center for Surveillance of Bacterial Resistance, Hefei, China; ^3^Institute of Bacterial Resistance, Anhui Medical University, Hefei, China; ^4^Department of Basic Medical Sciences, Anhui Provincial Laboratory of Microbiology and Parasitology, Anhui Medical University, Hefei, China; ^5^Department of Infectious Diseases, The Chaohu Hospital of Anhui Medical University, Hefei, China

**Keywords:** hepatocellular carcinoma, combination therapy, PD-1 blockade, TACE, microwave ablation (MWA), liver transplantation (LT)

## Abstract

Hepatocellular carcinoma (HCC) is a common malignant tumor with high extent of invasiveness. Its invasion process is closely related to complex tumor microenvironment and microvascular characteristics. Recently, immune combined targeted therapy has been applied to patients, combination therapy program with better effect needs to be explored. Atezolizumab combined Bevacizumab regimen in phase III clinical trial IMbrave150 was approved by U.S. Federal Drug Administration (FDA) for HCC treatment. This program is mostly used for liver malignant tumors have failed other treatments. Patients in terminal stage, overall curative has an unsatisfactory effect, survival time of patients is limited. Therefore, seeking best plan for combined treatment to improve patient's life quality and survival rate are still one of the most important clinical difficulties. This report describes a 37-year-old male who suffered from HCC repeatedly relapsed after hepatectomy. The patient received transcatheter arterial chemoembolization (TACE), microwave ablation (MWA), targeted therapy, and other combined treatments, all showed poor treatment effects. He received liver transplantation (LT) after receiving PD-1 blockade combined targeted therapy, eventually died due to severe immune rejection. It's first case of an allogeneic liver transplantation patient who received PD-1 blockade and Lenvaxen combined therapy. PD-1 blockade treatment and clinical observations of this case were summarized.

## Introduction

Liver cancer has become the second largest tumor in China. Most liver cancers develop from chronic hepatitis B. As global incidence of liver cancer continues to rise, primary liver cancer has become sixth most common cancer in world and second leading cause of cancer deaths. Hepatocellular carcinoma (HCC) is the most common type of primary liver cancer, which accounts for 90% of all primary liver tumors (1–3).

Most common underlying causes of HCC worldwide are chronic liver disease and cirrhosis, which are mainly caused by hepatitis B, hepatitis C, and alcoholic liver disease (4, 5). Although relevant literature revealed hepatitis B virus (HBV) could cause HCC without causing liver cirrhosis, most patients still experienced progression of liver cirrhosis (6). American Association for the Study of Liver Diseases (AASLD) guidelines recommend to detest HCC in HBV patients.

HCC development is a complex and diversified systematic process, which includes inflammatory damage, liver cell necrosis, and regeneration. However, there is currently no precise molecular targets. Different individuals may have different molecular genetic characteristics, showing different biological behavior, prognosis, and responses to molecular targeted therapy and immunotherapy (7).

Previous studies found 5-year survival rate of HCC was <15% (8, 9). Clinical treatment of HCC mostly adopts combination therapy, effect remains to be explored. To determine best combination therapy plan, a various of factors need to be considered, such as stage and scope, individual differences and current status, potential liver function, conditions and complications of extrahepatic diseases, etc. Current treatments include surgical resection and microwave ablation (MWA). Moreover, cancer immunotherapy has been applied to improve the specificity and strength of the immune system against cancer. A series of studies showed PD-1 therapy could promote the body's effective immune response to cancer cells (10, 11). Recent studies have shown combination of PD-1 or PD-L1 inhibitors with anti-CTLa-4 drugs holds great effect, different immune checkpoints inhibitors (ICIs) elicit synergistic anti-tumor immune responses (12, 13). Pinato and Marron illustrate for first time the positive relationship between trAEs and outcome from treatment with ICI in HCC (14). However, therapeutic effect of PD-1 blockade among different individuals is not yet clear. The treatment of patients receiving follow-up liver transplantation (LT) is also unclear.

This case reported a male with HCC, who was treated with PD-1 combined with Lenvaxen and received LT 6 months later. Postoperative effect was limited. Furthermore, acute immune rejection occurred after operation, followed by multiple organ failure. Recently, Lenvatinib plus pembrolizumab was found to be a potential drug for HCC treatment through research studies (14), there is no enough information about the relationship between Lenvaxen combined with PD-1 immunotherapy and organ transplant. In this report, we aim to determine whether the occurrence of acute immune rejection is related to the application of PD-1.

## Case Report

### General Situation

Display according to timeline: HCC patient with history of hepatitis B for more than 20 years received partial liver resection in 2018. After operation, he was complicated by repeated intrahepatic tumor recurrence and metastasis. He received various combined treatments such as liver resection, tumor chemotherapy, transcatheter arterial chemoembolization (TACE), liver MWA therapy, and single-dose Lenvaxen targeted therapy, but the effect was limited. He received PD-1 immunotherapy for half a year, and then received the LT.

### Medical History

In October 2018, a 37-year-old Chinese man with a history of hepatitis B for more than 20 years was admitted to First Affiliated Hospital of Anhui Medical University due to liver lesions 3 days. No clinical features such as jaundice or spider nevi were found in patient. Enhanced computed tomography (CT) and several subsequent laboratory auxiliary examinations revealed space-occupying lesions in right lobe, calcification of left lobe, cysts in right kidney. Besides, he also provided results of Ultrasonography by an external hospital, suggesting a solid mass in right lobe of liver, a slightly thickened spleen, and a cyst in right kidney. Tumor markers showed levels of carcinoembryonic antigen (CEA), carbohydrate antigen199 (CA199), and HSP90a were higher than normal. He underwent right hepatic lobe resection and cholecystectomy in November, 2018. Postoperative pathology showed well-differentiated HCC with adjacent tissues suggested nodular cirrhosis (**Figures 2A,B**). Entecavir hydrate and fluorouracil were taken routinely for maintenance. After treatment, his recovery was satisfactory, indicators gradually returned to normal.

### Recurrence

**Figure 1:**
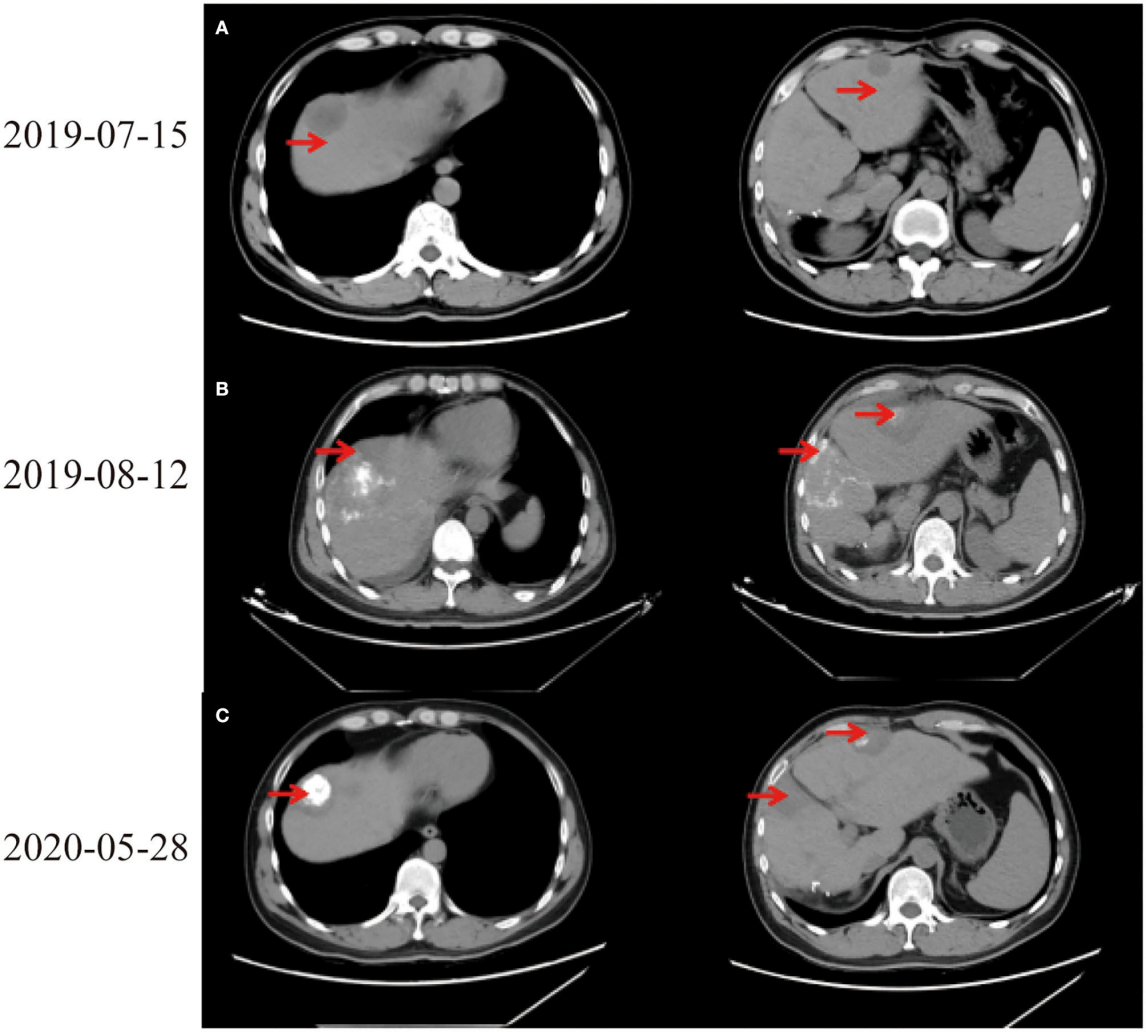
**(A–C)** The imagines of chest and abdomen by CT.

In July 2019, tumor biomarkers showed an increase of alpha fetoprotein (AFP) to 27.9 ng/ml. CT revealed intrahepatic recurrence, intrahepatic metastasis, and space-occupying lesions in left lobe of liver (Figure 1A). Computed tomography scans of chest, abdomen, and pelvis showed no evidence of extrahepatic metastasis. Laboratory indicators showed AFP 34.93 ng/ml, Alanine aminotransferase (ALT) 318 U/L, Aspartate aminotransferase (AST) 267 U/L, alkaline phosphatase (ALP) 73 U/L, and total bilirubin (TBIL) 34.5 μmol/L. After full discussion with patient and his family, LT was considered.

TACE via right hepatic artery and oral maintenance therapy with molecularly targeted drug orafenib tosylate and anti-hepatitis B virus drug entecavir hydrate were given. During the treatment, the AFP indicators were regularly reviewed and maintained within normal range. Orafenib tosylate was adjusted to lenvaxen half a month later due to severe drug side effects, including obvious peeling of hand and foot skin, inability to hold objects or walk. After adjustment, skin damage gradually healed.

**Figure 2:**
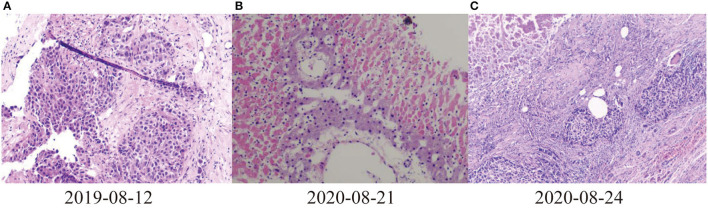
Pathological results of liver. **(A)** 2019-08-12 Puncture pathological diagnosis of poorly differentiated carcinoma with necrosis, hepatocellular carcinoma recurrence (occupying biopsy of the left lobe of the liver), **(B)** 2020-08-21Most of the liver tissues of the puncture pathological section showed infarct-like changes, most of the liver cells were fatty degeneration, and a large number of inflammatory cells exuded in the portal area and liver sinusoids **(C)** 2020-08-24 Pathological and immunohistochemical results of liver of liver transplant recipients: differentiation in hepatocellular carcinoma, Hepatocyte **(–)**, GP3C (+), CDX-2 **(–)**, CK20 **(–)**, CD34 (+), CK7 **(–)**, CK19 (–), Ki-67 (40%, +).

On August 6th, 2019, he was to hospital for reexamination due to abdominal discomfort. CT showed multiple abnormal signals in liver (Figure 1B). He received percutaneous MWA of liver space-occupying lesions under ultrasound guidance on August 9th, 2019. His abdominal pain eased and continued to take entecavir hydrate after discharge. Considering multiple liver lesions of the patient, 240 mg PD-1 blockade infusion treatment was used every 3 weeks from August 23rd, 2019. Tumor intrahepatic metastasis recurred in May 2020. MWA and TACE were used in July. During this period, PD-1 blockade infusion was always maintained. He received LT, postoperative pathological immunohistochemical results showed Hepatocyte (–), GP3C (+), CDX-2 (–), CK20 (–), CD34 (capillary +), CK7 (–), CK19 (–), Ki-67 (40%, +) (Figure 2C). Severe immune rejection occurred within 20 h after surgery. His liver function and coagulation indicators gradually deteriorated: TBIL 61.50 μmol/L, ALT 1,099 U/L, AST 2,797 U/L, ALP 35 U/L, lactate dehydrogenase (LDH) 21,341 U/L, creatinine (Cre) 110.1 μmol/L, plasma Prothrombin time (PT) 27.6 s, plasma prothrombin time activity (PTA) 29.00%, international standardized ratio (INR) 2.56, activated partial thromboplastin time (APTT) 77.1 s, fibrinogen (FIB) content 1.00 g/L, plasma thrombin time (TT) 22.7 s, D-dimer 1.90 μg/ml, fibrin (proto) degradation product (FDP) 5.24 μg/ml. Multiple organ dysfunction syndromes occurred.

**Figure 3:**
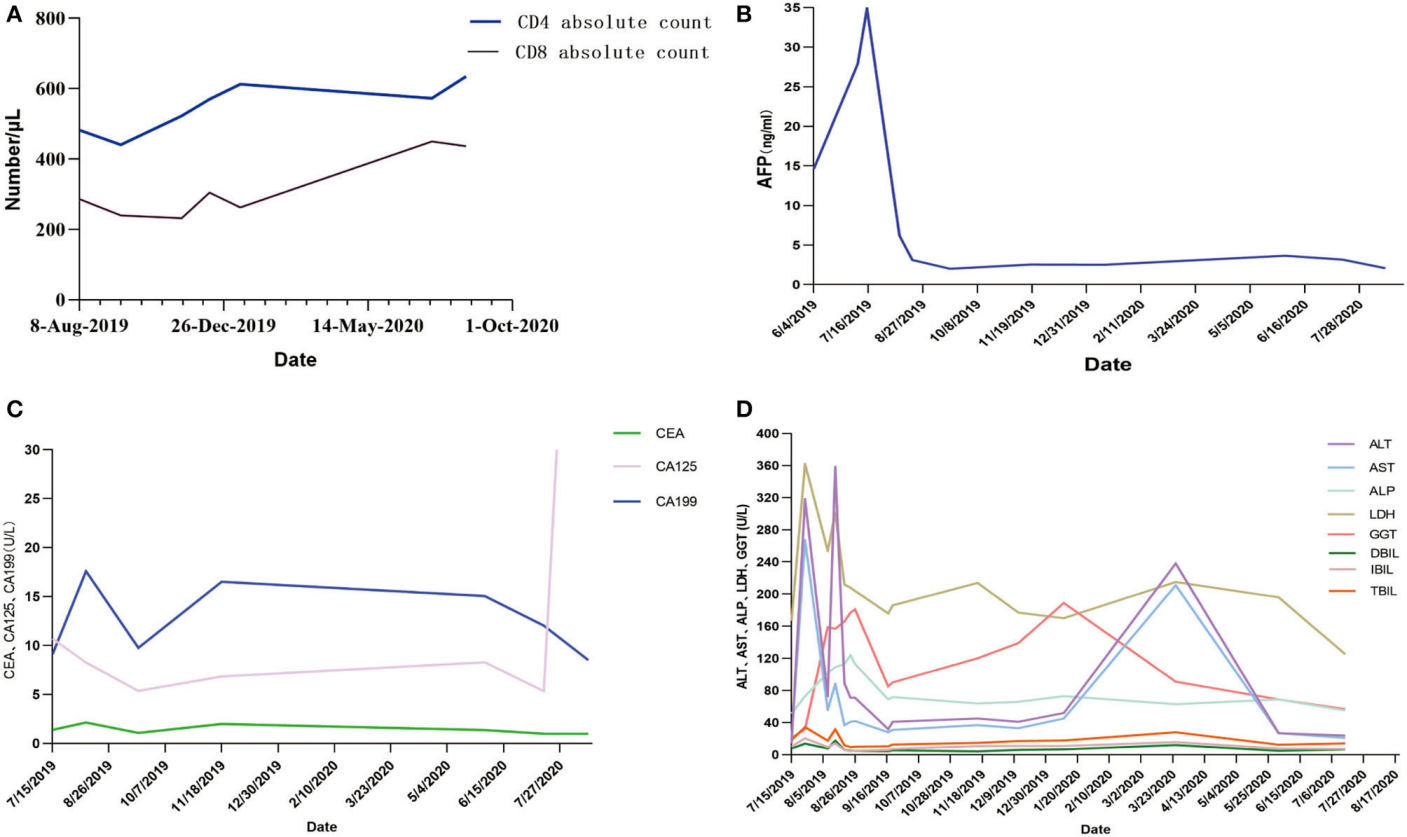
**(A–D)** AFP, CA199, and related liver indicators of the patient in the process of PD-1 blockade treatment. AFP, α-fetoprotein; CEA, carcinoembryonic antigen; CA199, Carbohydrate antigen199; CA125, carbohydrate antigen125; ALT, alanine aminotransferase; AST, aspartate aminotransferase; ALP, alkaline phosphatase; LDH, actate dehydrogenase; GGT, glutamyl transpeptidase; DBIL, direct bilirubin; IBIL, indirect bilirubin; TBIL, total bilirubin.

Some clinical data had been compared (Figure 3). When targeted drugs and PD-1 blockade were used, AFP level decreased significantly and remained within normal range, and CD4 cell and CD8 cell counts continued to increase slowly. This result showed combination therapy had obvious benefits to the patient. The patient's liver enzyme level also dropped significantly. In March 2020, periodic review revealed abnormally elevated transaminases in the patient. After hepatoprotective treatment, both ALT and AST decreased. The condition worsened again.

## Discussion

Clinical diagnosis of HCC and formulation of treatment plans mainly rely on early imaging tests and laboratory tests. Nowadays, there are still many controversies about best treatment plan. LT is still best choice in theory, which can completely cure liver disease. However, there are limitations due to many factors, such as shortage of recipient organs, immunosuppression and treatment costs. On the other hand, sparing transplantation is an option for patients with recurrent tumors or without further local treatments (15–17).

At present, immunotherapy has become first-line treatment for a variety of malignant tumors. Tumors can suppress immune response of the immune system by activating negative regulatory pathways related to immune homeostasis. Interaction between tumor cells and host immune system will promote immune escape, which will lead to spread, recurrence and metastasis of tumors. ICIs interfere with immunosuppressive signals to maintain immune homeostasis and enhance the body's anti-tumor immunity. However, its lack of specificity can lead to immune-related adverse events (irAEs), including non-target immune and inflammatory events, which are sometimes fatal (18).

This report described HCC case treated with lenvaxen and PD-1 blockade before and after allogeneic liver transplantation firstly. In process of PD-1 blockade treatment, the patient's AFP, CA199, and related liver indicators showed a trend of improvement (Figure 3). Life quality and living ability improved. This indicated lenvaxen combined with PD-1 blockade program had a certain effect on this patient.

PD-1 was reported to have an ability to increase incidence or severity of immune complications by enhance allogeneic T cell in organ transplantation patients (19–28). There were also some studies which showed PD-1 inhibitor immunotherapy was effective in LT patients (29, 30). In this report, the overall trend of CD4+ T and CD8+ T cells is also detected on rise with PD-1 blockade treatment (Figure 3C). Various indicators were improved during lenvaxen combined PD-1 blockade treatment. But the patient suffered severe immune rejection after subsequent LT and finally died. PD-1 therapy should be fully considered after organ transplantation. The best time for transplantation and immunotherapy, and screening the most suitable patients for such treatment needed further exploration. Further studies showed sirolimus could effectively relieve toxicity of PD-1 blockade (31). The patient was well tolerated during the course of PD-1 blockade, so this drug was not used.

**Figure 4:**
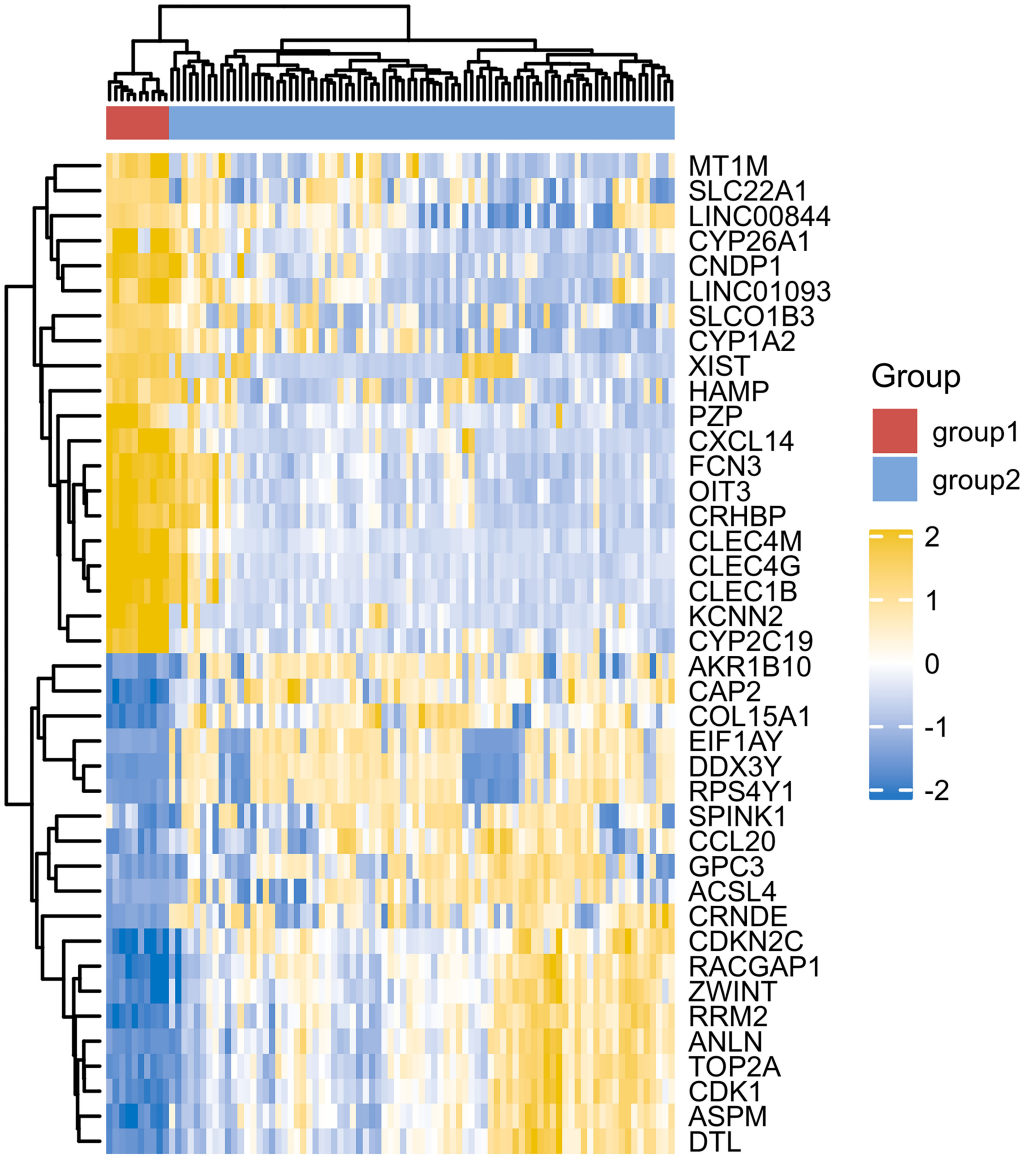
Differences in gene expression of HCC group (Group1) and healthy people (Group2).

**Figure 5:**
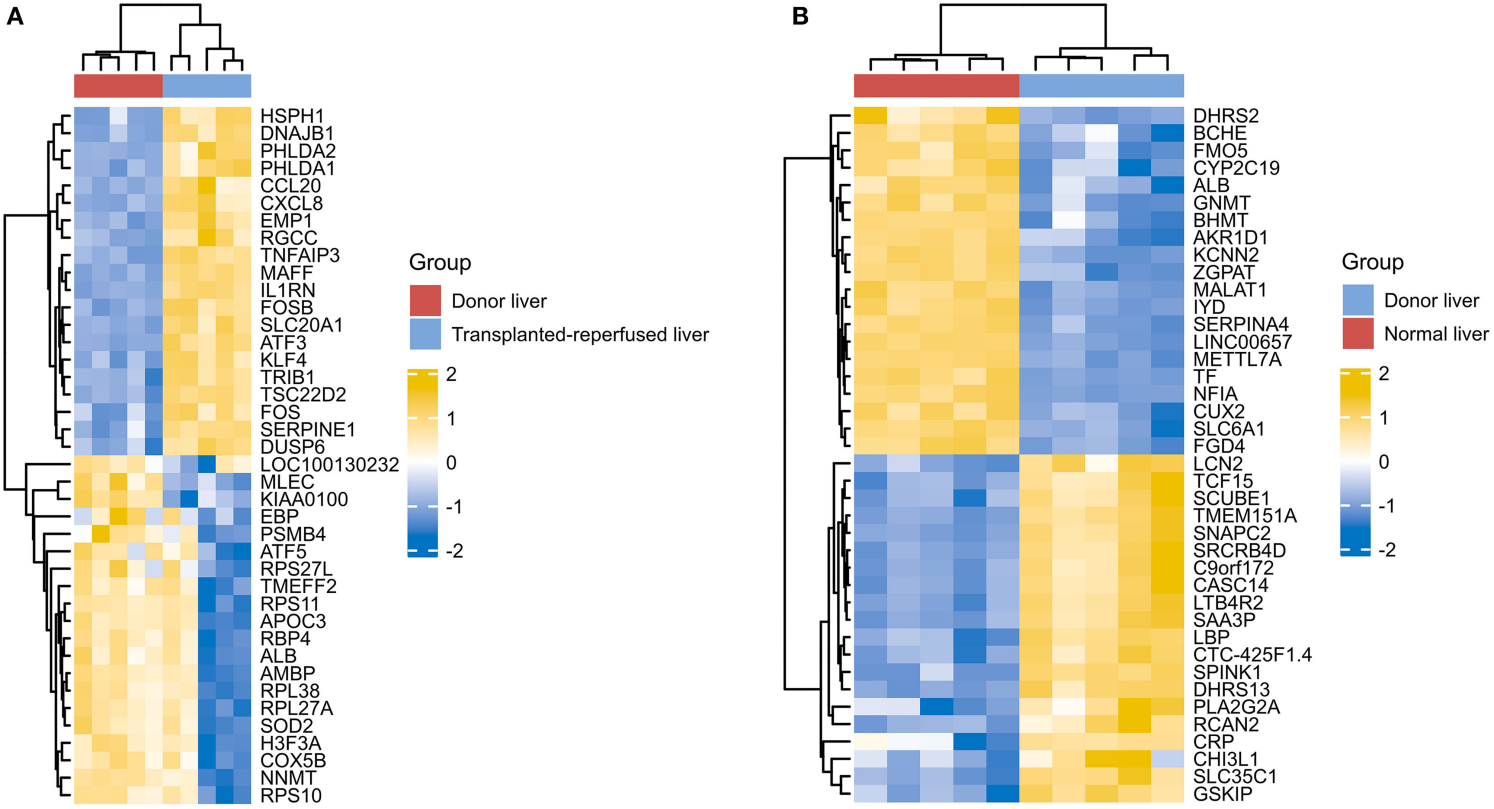
Differences of gene expression between the donor liver the healthy liver and the transplanted liver **(A,B)**.

However, due to differences of immunotherapy in different individuals and limitations of detection technology, even if his indicators show a significant improvement after immunotherapy, it's not possible to assess exact individual immunotherapy regimen and exact role of PD-1 mechanism. Moreover, to optimize comprehensive tumor treatment plan, R3.6.3 software (ComplexHeatmap) was used to analyze gene expression between patients with liver malignant tumors and healthy people from Gene Expression Omnibus (GEO) database. Highest and lowest expression of top 20 genes in HCC group (Group1) and healthy people (Group2) were observed (Figure 4). Results showed timely attention to whether gene expression of HCC patients is related to treatment effect of PD-1 monoclonal antibody can better guide the clinical formulation of treatment plans. In addition, we also analyzed gene expression between donor liver, healthy liver, and transplanted liver (Figure 5). It showed significant differences, and provide new detection targets for immunotherapy and comprehensive treatment of liver transplant patients (32, 33).

This study has some limitations: more specimens were not left in time for continuing trial, which will continue to be improved in subsequent studies. As number of patients with liver cancer is increasing, and immunosuppressant combined with targeted therapy has now become an important treatment for patients who cannot undergo liver resection or transplantation. This study provides experience in evaluating the period of LT and the suitability of LT for patients treated with immunotherapy in combination with targeted therapy, to conduct such studies as much as possible to avoid the occurrence of LT rejection after PD-1 application, *in vivo* and *in vitro* experiments can be conducted at a later stage to investigate the changes of indicators in perioperative period or after immunotherapy application in order to further investigate the optimal treatment protocol and population for HCC.

In this research, lenvaxen combined with PD-1 blockade was first compared before and after liver transplantation in patients with HCC. We provided a certain degree of experience for the clinical treatment of HCC and the application of PD-1 blockade in solid tumor transplantation. Further exploration of the exact role of gene and combined immunotherapy is needed to provide the necessary support for liver transplant patients waiting for donors and even organ transplantation, and to grasp the timing of immunotherapy and liver transplantation (34–37).

## Data Availability Statement

Publicly available datasets were analyzed in this study. This data can be found here: GEO database, (ID:200062232, 200014951).

## Ethics Statement

Ethical review and approval was not required for the study of human participants in accordance with the local legislation and institutional requirements. Written informed consent from the patients/participants or their legal guardian/next of kin was not required to participate in this study in accordance with the national legislation and the institutional requirements. Written informed consent was obtained from the patient for the publication of any potentially identifiable images or data included in this article.

## Author Contributions

JL and YL designed the study and thoroughly revised the manuscript. JY, MW, JC, LH, LY, XC, ZZ, and HL collected the data and analyzed the data. JY and MW drafted the manuscript. All authors contributed to the article and approved the submitted version.

## Funding

This study was supported by the National Natural Science Foundation of China (No. 81973983), the National Science and Technology Major Project (No. 2017ZX10204401), the Borrowing and Transferring Subsidy Project in 2019, Hefei (No. J2019Y04), Collaborative Tackling and Public Health Collaborative Innovation Project in Anhui Province (No. GXXT-2020-018), the Joint Construction Project of Clinical Medicine University and Hospital (No. 2021lcxk006), and Natural Science Research Project of Universities in Anhui Province (No. KJ2020A0176).

## Conflict of Interest

The authors declare that the research was conducted in the absence of any commercial or financial relationships that could be construed as a potential conflict of interest.

## Publisher's Note

All claims expressed in this article are solely those of the authors and do not necessarily represent those of their affiliated organizations, or those of the publisher, the editors and the reviewers. Any product that may be evaluated in this article, or claim that may be made by its manufacturer, is not guaranteed or endorsed by the publisher.
